# Progressive Changes in Lumbopelvic Alignment during the Three Month-Postpartum Recovery Period

**DOI:** 10.3390/ijerph19105807

**Published:** 2022-05-10

**Authors:** Mako Fukano, Kozo Aisaka, Sayaka Nose-Ogura, Tomoyuki Fujii, Suguru Torii

**Affiliations:** 1 College of Engineering, Shibaura Institute of Technology, Saitama-shi 337-8570, Japan; 2 Faculty of Sport Sciences, Waseda University, Tokorozawa-shi 359-1192, Japan; shunto@waseda.jp; 3 Hamada Hospital, Tokyo 101-0062, Japan; k_aisan@zaf.att.ne.jp; 4 Department of Obstetrics and Gynecology, The University of Tokyo, Tokyo 113-0033, Japan; o-sayaka@hotmail.co.jp; 5 Sanno Hospital, Tokyo 107-0052, Japan; tomoyuki.fujii@iuhw.ac.jp

**Keywords:** postpartum, lumbopelvic pain, lumbopelvic alignment, vaginal delivery, cesarean delivery, X-ray image

## Abstract

Pregnancy-related lumbopelvic pain is a common musculoskeletal problem, and postural changes are believed to be involved in these disorders. However, the lumbopelvic alignment changes in postpartum women remain unclear. This study aimed to determine whether there are changes in lumbopelvic alignment following vaginal or cesarean delivery and when these alignment changes occur after delivery. Thirty postpartum females (PP group) and 20 nulliparous female controls (CTL group) underwent anteroposterior, lateral pelvic, and lower-back X-ray in a static upright position. Digital radiographic images were analyzed and three radiographic variables, the pelvic incidence, pubic symphysis width, and sacral slope, were measured. The pubic symphysis width of the PP group was significantly larger immediately and one month after childbirth (PP group: 6.0 ± 1.1 mm (immediately), 5.0 ± 1.2 mm (one month); CTL group: 3.4 ± 0.4 mm; F = 31.79, *p* < 0.001). The sacrum slope in the PP group was significantly larger than in the CTL group 1 month after childbirth (PP group: 39.9 ± 6.6°; CTL group: 32.8 ± 5.1°; F = 2.59, *p* = 0.05). A two-way analysis of variance indicated no statistically significant main effects or interaction effects between the delivery modes on the pubic symphysis width or the sacrum slope. This study suggested that the course of lumbopelvic alignment progressed towards recovery for at least one month, and that these changes were independent of the delivery method.

## 1. Introduction

Pregnancy-related lumbopelvic pain, including pregnancy-related pelvic girdle pain (PPGP), pregnancy-related lower-back pain (PLBP), and their combination, are common musculoskeletal problems occurring during pregnancy and the postpartum period, with approximately 24.7% of postpartum women experiencing PPGP and/or PLBP [1]. These musculoskeletal disorders are thought to be due to a combination of mechanical and hormonal changes during pregnancy [2]. Recently, several studies have suggested that PPGP is more likely to be of biomechanical origin [3,4,5] than to be due to hormonal effects [6].

During pregnancy, considerable adaptation occurs in the joints within the lumbopelvic region to maintain equilibrium and ensure better joint load distribution. In the sagittal plane, the center of gravity typically shifts anteriorly in response to fetal growth [7]. There is also subsequent posterior inclination of the thoracic segments, resulting in greater anterior pelvic tilt and increased lumbar lordosis. During pregnancy and delivery, the cartilaginous joint at the pubic symphysis widens between the two pubic bones [8]. Although these adaptations during pregnancy are well known, very limited data regarding changes in lumbopelvic alignment in postpartum women are available, such as symphysis pubis width within 36 h of delivery [9], and pelvic alignment one month [8] and two months after delivery [10]. Furthermore, even less is known about the changes in lumbopelvic alignment occurring after vaginal and cesarean delivery. We hypothesized that postpartum women recover from this altered lumbopelvic alignment over time.

This information is critical for the development of evidence-based postpartum exercise programs to restore the pre-pregnancy state of the body, as well as for women’s plans to resume active fitness levels. Physical activity has a positive impact on the physical and mental health of postpartum women. In 2015, the American College of Obstetricians and Gynecologists recommended aerobic and strength conditioning exercises before, during, and after pregnancy for women with uncomplicated pregnancies [11]. However, the recovery process of altered lumbopelvic joints after childbirth is not well understood. Anatomical changes during and after pregnancy should be considered when developing and/or prescribing exercise programs, because inappropriate exercise intensity and/or load setting can lead to musculoskeletal injuries. The primary aims of this study were to determine whether there are differences in lumbopelvic alignment after vaginal and cesarean delivery and to determine how long after delivery these alignment changes at lumbopelvic sites occur. 

## 2. Materials and Methods

### 2.1. Participants

This study was approved by the local institutional ethics committee (application no.: 2017-230) and conducted in accordance with the guidelines set forth by the Declaration of Helsinki. The purpose, procedures, and risks of the study were explained to the participants, and written informed consent was obtained from each participant prior to their participation in the study.

Thirty postpartum females (PP group) and twenty nulliparous female controls (CTL group) participated in this study. The participants were recruited using advertisements at the university and in the hospital and volunteered to participate in the study. The CTL participants were nulliparous women of childbearing age who were free from lower-back and lower-extremity pain, a history of serious injuries, and any subjective symptoms interfering with daily life. The exclusion criterion was a history of serious injuries to the lower back, pelvic region, and lower extremities. A total 22 females were contacted as the CTL group, and 20 females were registered in order of arrival, except for two people who declined to participate and had lower-back pain.

### 2.2. X-ray Assessment

Each participant underwent anteroposterior, lateral pelvic, and low-back X-ray examination in a static upright position with their arms crossed on their chest (KXO-50S; Canon Medical Systems Corporation, Tochigi, Japan). In the PP group, X-ray images were acquired three times: within 5 days of childbirth (immediately), one month postpartum, and three months postpartum. The image resolution was 512 × 512 pixels.

**Figure 1:**
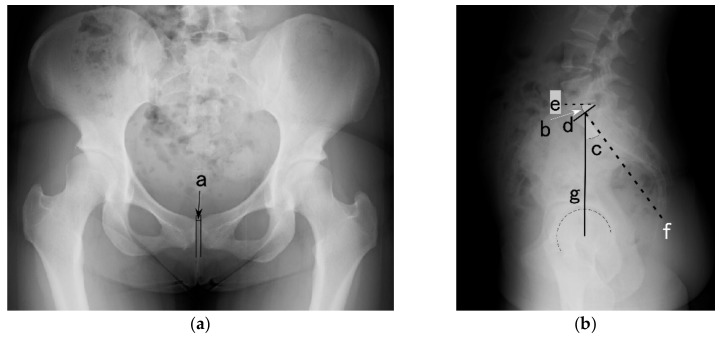
X-ray of the frontal image of pubic symphysis (**a**) and sagittal image of the lumbar region (**b**). a: pubic symphysis width, b: sacral slope, c: pelvic incidence, d: superior plate of sacrum, e: horizontal line, f: perpendicular line to the sacral plate at its midpoint, g: line connecting the midpoint of the sacral plate to the center of the femoral head.

Digital radiographic images were analyzed using graphic software (CANVAS 14; ACD Systems, Seattle, WA, USA). Three radiographic variables were measured: the pelvic incidence, pubic symphysis width, and sacral slope (Figure 1). The pelvic incidence was defined as the angle between the line perpendicular to the sacral plate at its midpoint and the line connecting this point to the axis of the femoral heads. The pelvic incidence is an anatomical parameter and a constant value [12]. The pubic symphysis width was defined as the minimum distance between the two parallel pubic bones. The sacral slope was defined as the angle between the superior plate of the sacral bone and the horizontal line. The sacral slope is a positional parameter of the pelvis, with a higher value indicating a more horizontal sacrum [12]. Image analyses and measurements were performed in a single-blinded manner from after image acquisition until group comparison.

### 2.3. Curl-Up Performance

All PP participants were asked to perform a curl-up and then questioned on whether they could perform a curl-up as an indicator of functional ability of the abdominal muscles at each session. Participants rated their performance based on their ability to raise their trunk during the curl-up exercise using the following scale (1–4): (*1) I do not remember how to contract the abdominal muscles; (2) I know how to contract the abdominal muscles; however, I am not able to contract the muscles; (3) I am able to contract the abdominal muscles; however, I am not able to perform a curl-up; (4) I am able to contract the abdominal muscles and perform a curl-up successfully.*

### 2.4. Statistical Analysis

All statistical analyses were performed using SPSS software (version 25; IBM Corp., Armonk, NY, USA). Differences in characteristics between the PP and CTL groups were determined using the independent two-tailed *t*-test. Two-way analysis of variance (ANOVA; delivery mode × time) (two-tailed) was performed to determine whether significant differences existed in terms of the pubic symphysis width and the sacrum slope between females who underwent vaginal delivery and cesarean delivery within the PP group at each time point, before comparison between PP participants and CTLs. One-way ANOVA (two-tailed) was also performed to test for differences in the pubic symphysis width and sacral slope between the PP and CTL groups at each time point. If statistical significance was obtained, post hoc tests (Bonferroni) were performed. Statistical significance was set at *p* < 0.05. The data are presented as mean ± standard deviation.

## 3. Results

There were no statistically significant differences in the age, height, weight (pre-pregnancy weight for the postpartum females), or pelvic incidence between the two groups. The demographic data of the PP group and CTL group are shown in Table 1.

The two-way ANOVA indicated no statistically significant difference in the main effect or the interaction effect between the delivery modes on the pubic symphysis width and the sacrum slope; thus, the following describes the results of the comparison between the PP and CTL groups. The pubic symphysis width of the PP group was significantly larger immediately and one month after childbirth (F = 31.79, *p* < 0.001). The sacral slope of the PP group was significantly larger than that of the CTL group one month after childbirth (F = 2.59, *p* = 0.05) (Table 2).

**Figure 2:**
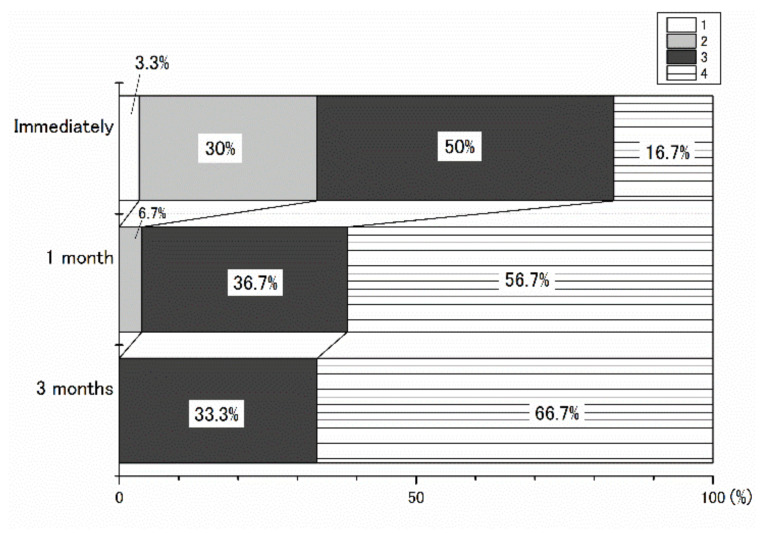
Breakdown of the curl-up performance of postpartum participants immediately, one month, and 3 months after childbirth. The scale 1–4 indicates: *(1) I do not remember how to contract the abdominal muscles; (2) I know how to contract the abdominal muscles, however, I am not able to contract the muscles; (3) I am able to contract the abdominal muscles, however, I am not able to perform a curl-up; (4) I am able to contract the abdominal muscles, and perform a curl-up successfully*. Participants chose the closest number describing their curl-up performance.

Figure 2 shows the breakdown of the curl-up performance scale. Only immediately after childbirth, there were participants who could not remember how to contract their abdominal musles (scale 1). The percentage of participants who knew how to contract their abdominal muscles but were unable to actually contract them (scale 2) decreased over time after childbirth and was 0% at three months after childbirth. The percentage of participants who were able to contract their abdominal muscles but were unable to perform curl-ups (scale 3) was 50% immediately after childbirth, 36.7% at one month postpartum, and 33.3% at three months postpartum. The percentage of participants who were able to contract their abdominal muscles and perform a curl-up successfully (scale 4) was 16.7% immediately after childbirth. This percentage subsequently increased to 56.7% at one month and to 66.7% at three months after childbirth.

## 4. Discussion

We investigated changes in the alignment of the lumbopelvic region of women after childbirth. We also investigated whether the measured parameters differed according to the delivery mode. Our results demonstrated that the pubic symphysis width of the PP group was significantly larger immediately and one month after childbirth; however, the distance decreased and was comparable to that of the CTL group at three months postpartum. The sacral slope angle was significantly larger at one month after childbirth; however, no statistically significant differences were observed immediately or at three months after childbirth. Both parameters were comparable in the groups divided by the delivery mode. This indicates that the biomechanical demands during pregnancy cause these lumbopelvic changes, since the lumbopelvic alignment is not influenced by the mode of delivery.

The pubic symphysis width immediately after childbirth in this study was 6.0 mm, irrespective of the delivery mode, and was comparable with that reported in a previous study by Aydin et al., in which the average pubic symphysis width was 7.09 mm using X-ray radiographs and 6.07 mm using three-dimensional transperineal ultrasonography within 36 h of delivery [9]. The lack of difference in pubic symphysis width with different delivery modes was consistent with a study by Agten et al., who reported that the distension of the pubic joint and fluid within the first postpartum week were similar among vaginal and cesarean deliveries, as analyzed by MRI [13]. We found that at 3 months postpartum, the width of the once-widened pubic symphysis had decreased and, subsequently, no statistically significant difference was observed. This result suggests that the progressive recovery of the pubic joint initiated at one month after childbirth. Additionally, a previous MRI study reported that bone marrow edema was observed in the pubic bones of 55% of postpartum women at 1 month after childbirth [14]. Therefore, there may be a time difference between the reduction in pubic joint width and the subsidence of pubic joint inflammation. Therefore, careful attention should be paid to the resumption of physical activity even after three months, since a lapse of three months post-delivery does not necessarily mean complete recovery of the pubic joint.

A larger sacral slope was only observed at 1 month after childbirth, although the angle was comparable to that in the CTL group immediately after childbirth. Catena et al. reported that the centers of mass of the torsos in postpartum women, which had shifted anteriorly during pregnancy, returned to their original position after childbirth, and that the women assumed a standing posture with the hip joint extended; nonetheless, the lumbar extension angle was increased 4–8 weeks after childbirth [7]. The authors assumed that this change was required to accommodate the women’s postpartum breast weight. Another contributing factor in increased anterior pelvic tilt may be abdominal muscle disfunction. Sagittal lumbopelvic alignment is related to the functional ability of the abdominal wall muscles. A previous study on the morphological and functional recovery of the abdominal muscles reported that internal abdominal oblique (IO) thickness decreased during pregnancy. Furthermore, the thinner IO muscle persisted for six months after delivery, while decreased transverse abdominis contractile function persisted for four months postpartum [15]. Our results suggest that the ability of the abdominal muscles to stabilize the pelvis may remain compromised for at least until 1 month after delivery; over 40% of the postpartum women in this study were not able to successfully execute curl-ups at this time.

This study has certain limitations that should be considered. The sample size was relatively small because of the risk of radiation exposure to the participants, considering that our primary purpose was to determine lumbopelvic alignment changes after delivery. A larger sample size is required to examine the association between lumbopelvic alignment and the incidence of pain and other symptoms.

## 5. Conclusions

This study suggested that lumbopelvic alignment progresses towards recovery for at least one month following childbirth. Further, after delivery, there is a possible discrepancy between physiological changes and subjective recovery in postpartum women. Gausel et al. reported that subjective substantial recovery from PPGP occurred within 2 weeks of delivery for many postpartum women [16]. Postpartum women, therefore, should seek proper counseling from clinicians and/or guidance from sports professionals prior to resuming physical activity and/or exercise.

## Figures and Tables

**Table 1:** Demographic data of the perinatal and control groups.

Group	Postpartum		Control		
	(*n* = 30)		(*n* = 20)		
Variables	Mean	SD	Mean	SD	*p*-Value
Height (cm)	157.6	4.9	157.6	4.4	0.98
Weight (kg) *^1^	53.8	9.9	51.3	6.8	0.76
Age (years)	33.0	4.4	32.0	10.6	0.77
Pelvic incidence (deg)	51.1	8.8	46.9	8.7	0.11

*^1^ Weight before pregnancy for the perinatal group; SD, standard deviation.

**Table 2:** Comparison of the pubic symphysis width and sacrum slope between the postpartum and control groups.

Group	Postpartum						Control	
	Immediately		1 Month		3 Months		(*n* = 20)	
Variables	Mean	SD	Mean	SD	Mean	SD	Mean	SD
Pubic symphysis width (mm)	6.0	1.1 *	5.0	1.2 *	3.9	1.1	3.4	0.4
Sacral slope (deg)	37.8	5.9	39.9	6.6 *	37.8	6.7	34.8	5.1

* *p* < 0.05 (vs. control group); SD, standard deviation.

## Data Availability

The data presented in this study are available on request from the corresponding author.
